# Supplementary material for: An alternative EGFR activation by patient-derived R252C mutation promotes cancer progression
Source: Nat Commun. 2026 Jan 21;17:1902. doi: 10.1038/s41467-026-68699-4 (PMC12923773; doi:10.1038/s41467-026-68699-4)
Supplement: Supplementary file 1 — Supplementary Information [file 41467_2026_68699_MOESM1_ESM.pdf]

Supplementary Information

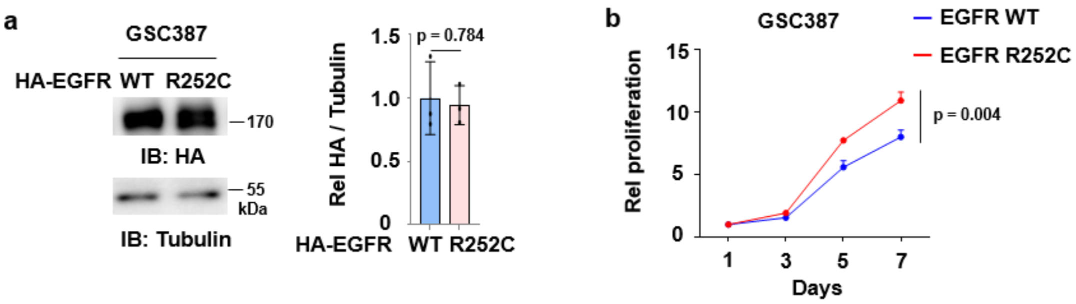

Supplementary Fig. 1: EGFR R252C promotes tumor cell proliferation. Related to Fig. 1.

a. GSC387 cells were infected with lentivirus stably expressing HA-EGFR WT or R252C. Cells were harvested and subjected to immunoblotting analyses. Semi-quantitative scoring was also carried out. Rel, relative.

b. Cells in (a) were cultured in complete media supplemented with 10% fetal bovine serum (FBS). Cell proliferation assay was performed with cells in (a). Relative cell proliferation of GSC387 cells expressing EGFR WT or R252C was normalized to the day 1 value.

a,b. Data represent the mean ± s.d. of three biologically independent experiments (unpaired, two-tailed t-test).

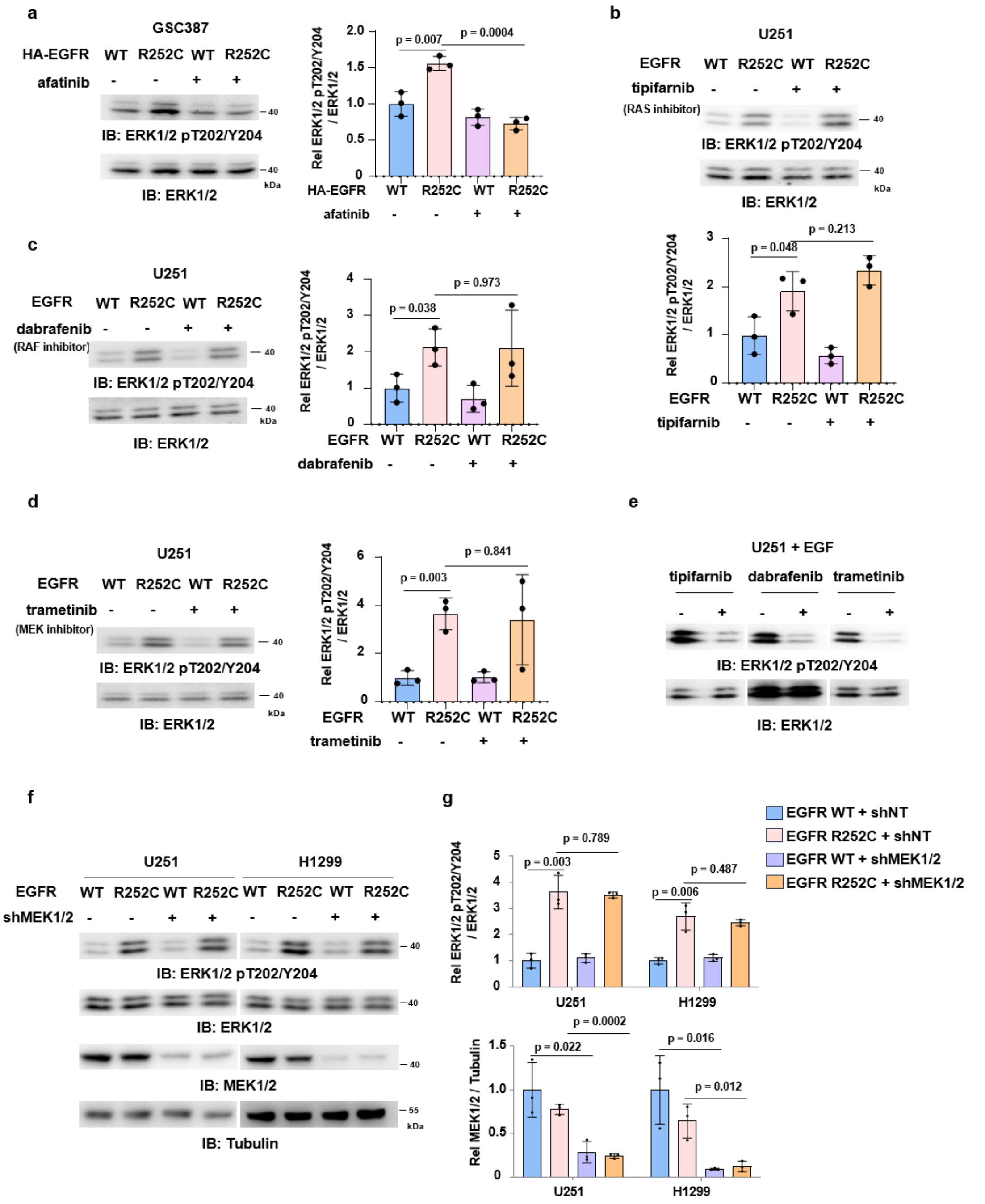

Supplementary Fig. 2: EGFR R252C activates ERK1/2 independent of RAS/RAF/MEK pathway. Related to Fig. 2.

a. GSC387 cells stably expressing HA-EGFR WT or R252C were cultured in complete media containing 10% FBS. Cells were treated with either DMSO or 10 μM Afatinib for 6 hr prior to harvesting for immunoblotting analysis. Semi-quantitative scoring was carried out. The phosphorylated-ERK1/2 to ERK1/2 ratio was quantified and normalized to the DMSO-treated EGFR WT group.

b,c,d. U251 cells harboring either EGFR WT or EGFR R252C were cultured in complete media containing 10% FBS. Cells were treated with 10 μM RAS inhibitor (tipifarnib), 10 μM RAF inhibitor (dabrafenib), or 10 μM MEK inhibitor (trametinib) for 6 hr, respectively. Cells were harvested and subjected to immunoblotting analyses. Semi-quantitative scoring was also carried out, and the phosphorylated-ERK1/2 to ERK1/2 ratio was quantified and normalized to the DMSO-treated EGFR WT group.

e. U251 cells were serum-starved for 24 hr and then treated with 10 μM RAS inhibitor (tipifarnib), 10 μM RAF inhibitor (dabrafenib), or 10 μM MEK inhibitor (trametinib) for 6 hr, followed by stimulation with 10 ng/ml EGF for 30 min. Cells were harvested and subjected to immunoblotting analyses.

f,g. MEK1/2 were depleted in U251 or H1299 cells harboring either EGFR WT or EGFR R252C. Cells were cultured in complete media supplemented with 10% FBS and subsequently harvested for immunoblotting analysis. The samples derive from the same experiment but different gels for phospho-ERK1/2, ERK1/2， and another for MEK1/2, Tubulin were processed in parallel. Semi-quantitative scoring was also carried out. The phosphorylated-ERK1/2 to ERK1/2 and MEK1/2 to Tubulin ratios were quantified and normalized to the shNT-expressing EGFR WT cells.

a-d,g. Data represent the mean ± s.d. of three biologically independent experiment (unpaired, two-tailed t-test).

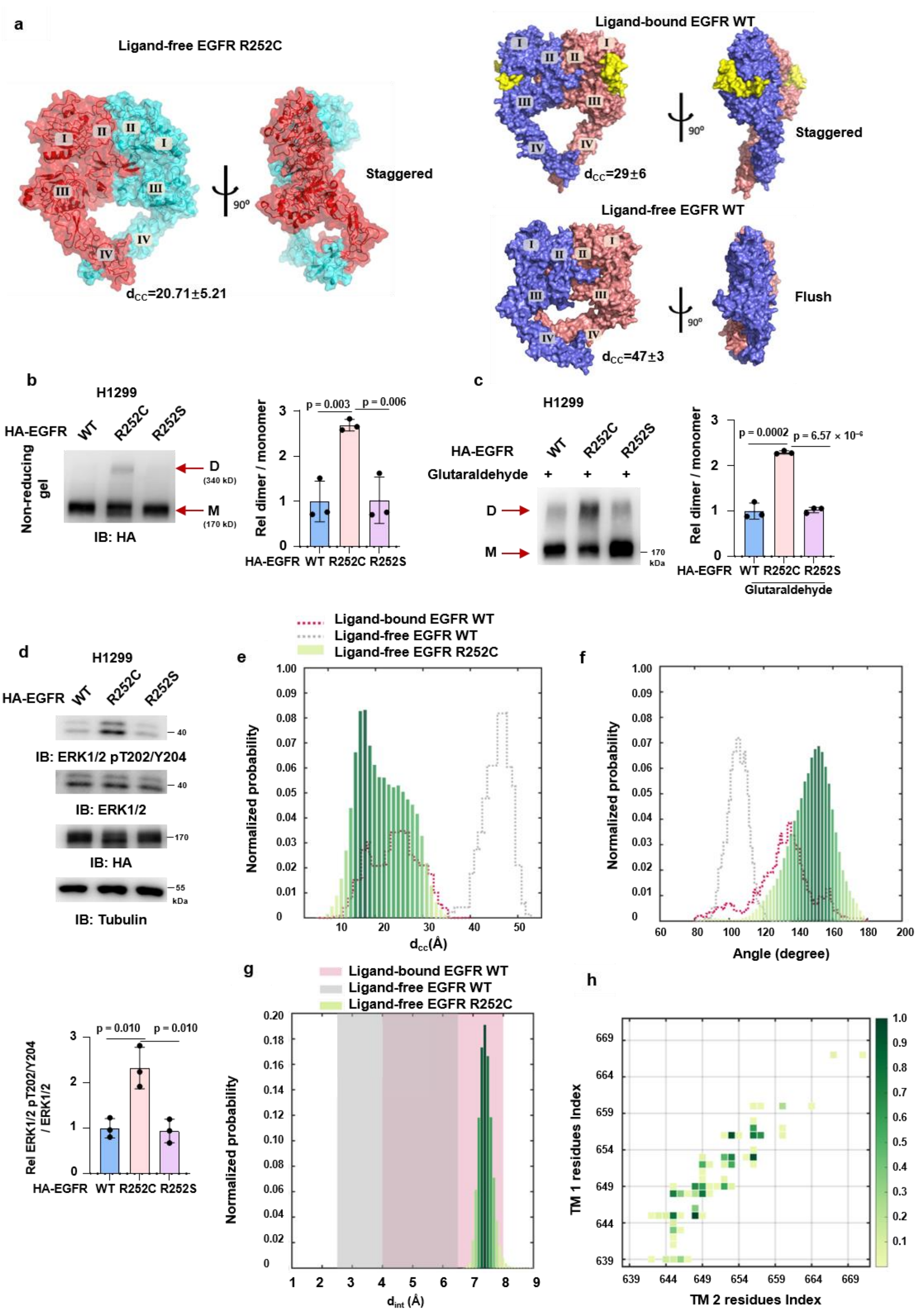

Supplementary Fig. 3: Structural basis of EGFR R252C-dependent ERK1/2 activation. Related to Fig. 3.

a. Conformations of the ligand-free EGFR R252C extracellular dimer (left panel), ligand-bound and ligand-free EGFR WT extracellular dimer (right panel). The conformations

in ligand-free EGFR WT and ligand-bound EGFR WT were obtained from the work of Arkhipov et al. (Cell 152, 557-569).

b. H1299 cells were transfected with HA-EGFR WT, R252C, or R252S. Cells were cultured in complete media supplemented with 10% FBS and subsequently harvested for non-reducing SDS-PAGE. Semi-quantitative scoring was also carried out. The dimer to monomer ratio was quantified and normalized to the EGFR WT cells.

c. H1299 cells transfected with HA-EGFR WT, R252C, or R252S were cultured in complete media supplemented with 10% FBS. Cells were cross-linked with glutaraldehyde and subsequently harvested for immunoblotting analysis. Semi-quantitative scoring was also carried out. The dimer to monomer ratio was quantified and normalized to the EGFR WT cells.

d. H1299 cells were transfected with HA-EGFR WT, R252C, or R252S. Cells were cultured in complete media supplemented with 10% FBS and subsequently harvested for immunoblotting analyses. Semi-quantitative scoring was also carried out. The phosphorylated-ERK1/2 to ERK1/2 ratio was quantified and normalized to the EGFR WT group.

e. The d_{cc} of ligand-free R252C EGFR, ligand-free EGFR WT, and ligand-bound EGFR WT are displayed. The values of d_{cc} and angles were colored in green, gray, and salmon for ligand-free EGFR R252C, ligand-free EGFR WT, and ligand-bound EGFR WT, respectively.

f. “Staggered’’ conformation of the extracellular dimers can be characterized by the angle formed by the Cα of I214 and P228 of one subunit and P228 of the other, following the definition of Arkhipov et al. (e,f) The values of d_{cc} and angle in ligand-free EGFR WT and ligand-bound EGFR WT were obtained from the work of Arkhipov et al. (Cell 152, 557-569).

g. The center-of-mass distance between the GxxxG-like motif of the two transmembrane helices is plotted. The values of d_{int} and contacts were colored in green, gray, and salmon for ligand-free EGFR R252C, ligand-free EGFR WT, and ligand-bound EGFR WT, respectively. The values of d_{int} in ligand-free EGFR WT and ligand-bound EGFR WT were obtained from the work of Arkhipov et al. (Cell 152, 557-569).

h. The residue-residue contact between the two helices in ligand-free EGFR R252C, where the intensity represents the fraction of simulation time in which a contact is maintained.

b-d. Data represent the mean ± s.d. of three biologically independent experiments (unpaired, two-tailed t-test).

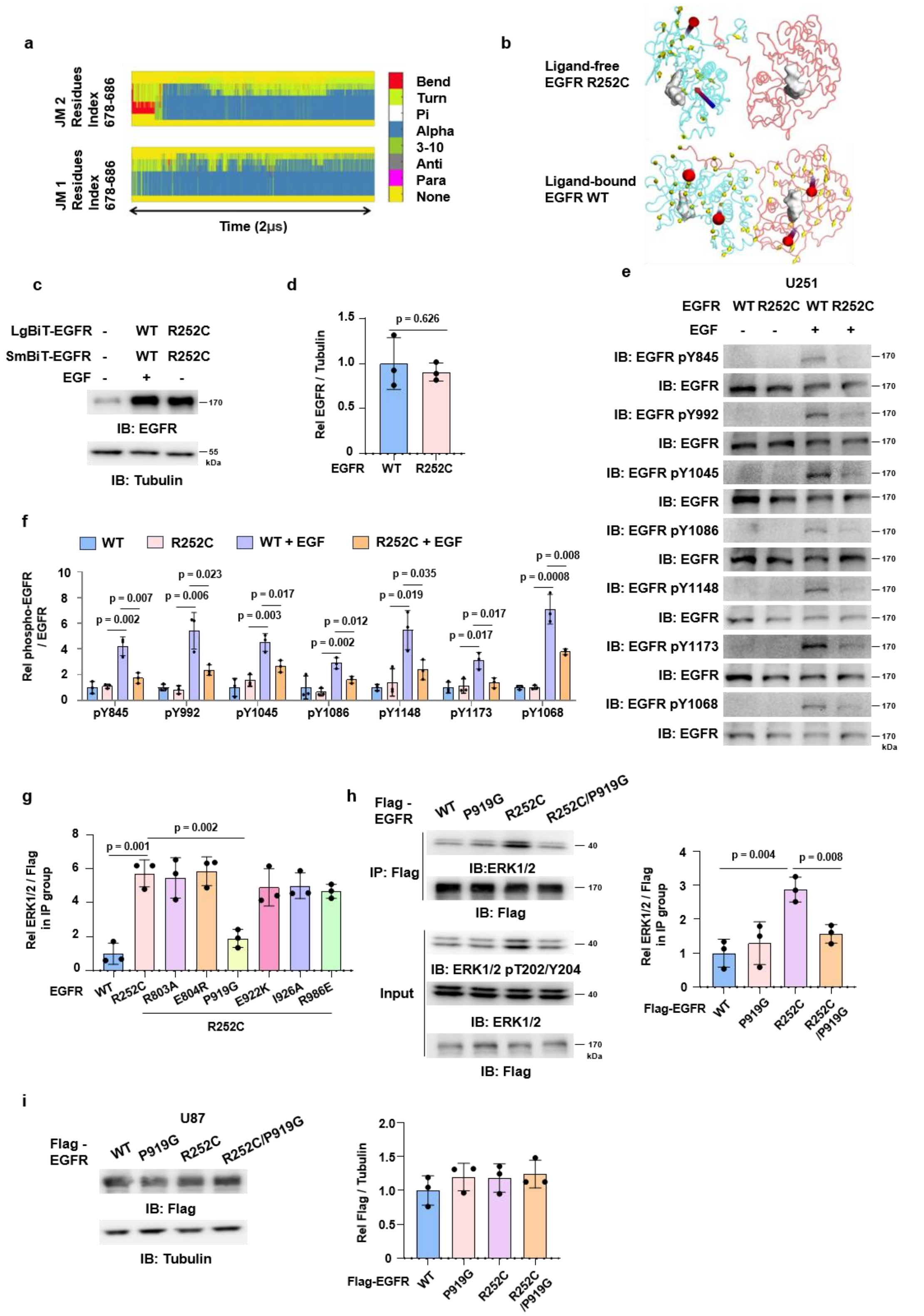

Supplementary Fig. 4: Structural basis of EGFR R252C-dependent ERK1/2 activation. Related to Fig. 3.

a. Helicity of the JM segments in ligand-free EGFR R252C based on the data from the simulation of the ECD-TM-JM construct.

b. The principal component analysis (PCA) of ligand-free EGFR R252C and ligand-bound EGFR WT. The active site was shown in surface and colored in white. The representative principal component of different lobe was shown in arrow and colored in arrow with red front and blue end.

c,d. EGFR WT or R252C-SmBiT together with EGFR WT or R252C-LgBiT and control Renilla plasmid were used to transfect HEK293T cells. Cells transfected with EGFR WT were serum-starved for 24 hr and then stimulated with 10 ng/ml EGF for 30 min. Cells were harvested and subjected to immunoblotting analyses. Semi-quantitative scoring was also carried out. The EGFR to Tubulin ratio was quantified and normalized to the EGF-treated EGFR WT group (d).

e,f. U251 cells expressing EGFR WT or EGFR R252C were either maintained in complete media or subjected to serum starvation for 24 hr followed by stimulation with 10 ng/ml EGF for 30 min. All cells were then harvested for immunoblotting analysis (e). The samples derive from the same experiment but different gels for EGFR pY845, EGFR, another for EGFR pY992, EGFR, another for EGFR pY1045, EGFR, another for EGFR pY1086, EGFR, another for EGFR pY1148, EGFR, another for EGFR pY1173, EGFR, and another for EGFR pY1068, EGFR were processed in parallel. Semi-quantitative scoring was also carried out, and the phosphorylated-EGFR to EGFR ratio was quantified and normalized to the unstimulated EGFR WT cells (f).

g. Immunoprecipitation assay was performed with HEK293T cells stably expressing Flag-EGFR WT, R252C or indicated mutants. Semi-quantitative scoring was carried out. The ERK1/2 to Flag ratio was quantified and normalized to the EGFR WT cells.

h. Immunoprecipitation assay was performed with HEK293T cells stably expressing Flag-EGFR WT, P919G, R252C or R252C/P919G. Semi-quantitative scoring was carried out, and the ERK1/2 to Flag ratio was quantified and normalized to the EGFR WT cells.

i. Immunoblotting assay was performed with U87 cells stably expressing Flag-EGFR WT, P919G, R252C or R252C/P919G. Semi-quantitative scoring was carried out. The Flag to Tubulin ratio was quantified and normalized to the EGFR WT cells.

d,f-i. Data represent the mean ± s.d. of three biologically independent experiments (unpaired, two-tailed t-test).

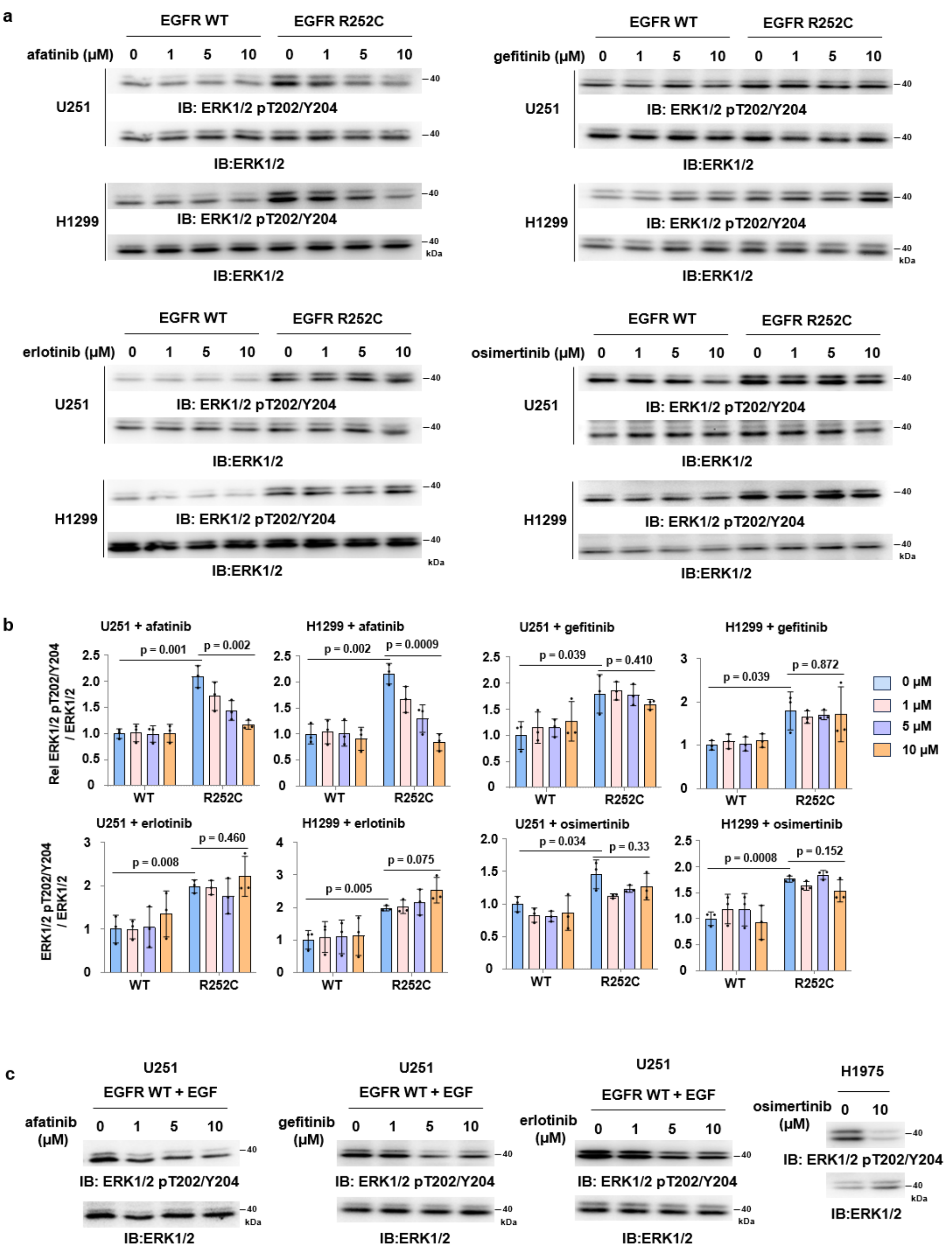

Supplementary Fig. 5: Afatinib inhibits EGFR R252C-induced ERK1/2 activation. Related to Fig. 4.

a,b. U251 or H1299 cells harboring either EGFR WT or EGFR R252C were maintained in complete media and treated with different doses of afatinib, gefitinib, erlotinib, or osimertinib for 1 hr. Cells were harvested and subjected to immunoblotting analyses. Semi-quantitative scoring was also carried out, and the phosphorylated-ERK1/2 to ERK1/2 ratio was quantified

and normalized to vehicle-treated EGFR WT cells. Data represent the mean ± s.d. of three biologically independent experiments. Unpaired, two-tailed t-test(b).

c. U251 cells were serum-starved for 24 hr and then treated with afatinib, gefitinib, or erlotinib for 1 hr, followed by stimulation with 10 ng/ml EGF for 30 min. H1975 cells were cultured in complete media containing 10% FBS and treated with osimertinib for 1 hr. Cells were harvested and subjected to immunoblotting analyses.

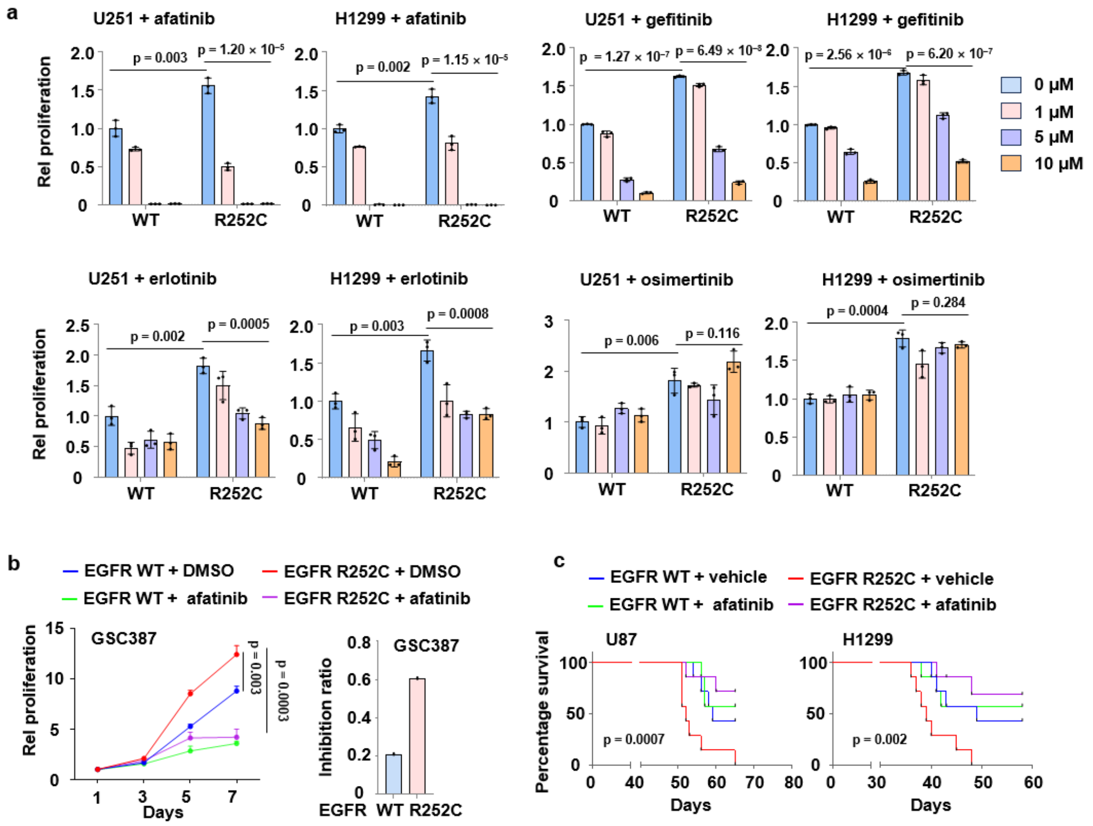

Supplementary Fig. 6: Afatinib inhibits EGFR R252C-driven tumor cell proliferation and tumor progression. Related to Fig. 4.

a. U251 or H1299 cells harboring either EGFR WT or EGFR R252C were maintained in complete media and treated with different doses of afatinib, gefitinib, erlotinib, or osimertinib. Cell proliferation was assessed at day 7. Relative proliferation was calculated using vehicle-treated EGFR WT cells as the control.

b. GSC387 cells stably expressing HA-EGFR WT or R252C were maintained in complete media and treated with either vehicle (DMSO) or 1 μM Afatinib. Cell proliferation assay was performed in these cells. Relative cell proliferation of each group was normalized to its respective day 1 value. The inhibition ratio of cell proliferation by afatinib was calculated at day 7.

c. (left) Luciferase-expressing U87 cells with or without EGFR R252C were intracranially injected into randomized athymic nude mice (seven mice per group). After 35 days, Afatinib was administered via oral gavage (10 mg/kg body weight) for a duration of 14 days. Kaplan-Meier survival analysis was performed. Data represent the mean ± s.d. of seven mice.

(right) Luciferase-expressing H1299 cells with or without EGFR R252C were injected into the left lung of randomized athymic nude mice (seven mice per group). After 21 days, Afatinib was administered via oral gavage (10 mg/kg body weight) for a duration of 14 days. Kaplan-Meier survival analysis with two-tailed log-rank test was performed. Data represent the mean ± s.d. of seven mice.

a,b. Data represent the mean ± s.d. of three biologically independent experiments (unpaired, two-tailed t-test).
