# Supplementary material for: An alternative EGFR activation by patient-derived R252C mutation promotes cancer progression
Source: Nat Commun. 2026 Jan 21;17:1902. doi: 10.1038/s41467-026-68699-4 (PMC12923773; doi:10.1038/s41467-026-68699-4)
Supplement: Supplementary file 2 — Reporting Summary [file 41467_2026_68699_MOESM2_ESM.pdf]

nature portfolio

Corresponding author(s): Weiwei Yang

Last updated by author(s): Dec 6, 2025

Reporting Summary

Nature Portfolio wishes to improve the reproducibility of the work that we publish. This form provides structure for consistency and transparency in reporting. For further information on Nature Portfolio policies, see our Editorial Policies and the Editorial Policy Checklist.

Statistics

For all statistical analyses, confirm that the following items are present in the figure legend, table legend, main text, or Methods section.

n/a	Confirmed	
☐	☒	The exact sample size (*n*) for each experimental group/condition, given as a discrete number and unit of measurement
☐	☒	A statement on whether measurements were taken from distinct samples or whether the same sample was measured repeatedly
☐	☒	The statistical test(s) used AND whether they are one- or two-sided<br>*Only common tests should be described solely by name; describe more complex techniques in the Methods section.*
☒	☐	A description of all covariates tested
☒	☐	A description of any assumptions or corrections, such as tests of normality and adjustment for multiple comparisons
☐	☒	A full description of the statistical parameters including central tendency (e.g. means) or other basic estimates (e.g. regression coefficient) AND variation (e.g. standard deviation) or associated estimates of uncertainty (e.g. confidence intervals)
☐	☒	For null hypothesis testing, the test statistic (e.g. *F*, *t*, *r*) with confidence intervals, effect sizes, degrees of freedom and *P* value noted<br>*Give P values as exact values whenever suitable.*
☒	☐	For Bayesian analysis, information on the choice of priors and Markov chain Monte Carlo settings
☒	☐	For hierarchical and complex designs, identification of the appropriate level for tests and full reporting of outcomes
☒	☐	Estimates of effect sizes (e.g. Cohen's *d*, Pearson's *r*), indicating how they were calculated

Our web collection on statistics for biologists contains articles on many of the points above.

Software and code

Policy information about availability of computer code

Data collection	Immunoblotting data: Tanon-5200 Chemiluminescent Imaging System (version 1.0)<br>Cell Proliferation data: BioTek EON (version 1.0)<br>Colony formation data: Tanon-5200 Chemiluminescent Imaging System (version 1.0)<br>Bioluminescence imaging data: Tanon-5200 Chemiluminescent Imaging System (version 1.0)
Data analysis	Densitometry analysis ofthe immunoblots: Image J (version 1.52a)<br>Colony counting: Image J (version 1.52a)<br>Statistical analysis: GraphPad Prism (version 8.0)<br>Bioluminescence imaging analysis: Tanon-5200 Chemiluminescent Imaging System (version 1.0)

For manuscripts utilizing custom algorithms or software that are central to the research but not yet described in published literature, software must be made available to editors and reviewers. We strongly encourage code deposition in a community repository (e.g. GitHub). See the Nature Portfolio guidelines for submitting code & software for further information.

Data

Policy information about availability of data

All manuscripts must include a data availability statement. This statement should provide the following information, where applicable:

- Accession codes, unique identifiers, or web links for publicly available datasets
- A description of any restrictions on data availability
- For clinical datasets or third party data, please ensure that the statement adheres to our policy

Source data are provided with this paper.

Research involving human participants, their data, or biological material

Policy information about studies with human participants or human data. See also policy information about sex, gender (identity/presentation), and sexual orientation and race, ethnicity and racism.

Reporting on sex and gender	Not applicable.
Reporting on race, ethnicity, or other socially relevant groupings	Not applicable.
Population characteristics	Not applicable.
Recruitment	Participants were not recruited.
Ethics oversight	The patient described in this study was not actively enrolled by our research team. Clinical data were retrospectively collected through medical record review following institutional protocols. Ethical approval for the human participant was approved by the Ethics Committee in Clinical Research (ECCR) of the First Affiliated Hospital of Wenzhou Medical University and the study was performed in accordance with the approved protocol. Informed consent was obtained from the patient's family for both research and publication of de-identified data included in this article. No compensation was provided to the participant.

Note that full information on the approval of the study protocol must also be provided in the manuscript.

Field-specific reporting

Please select the one below that is the best fit for your research. If you are not sure, read the appropriate sections before making your selection.

☒ Life sciences ☐ Behavioural & social sciences ☐ Ecological, evolutionary & environmental sciences

For a reference copy of the document with all sections, see nature.com/documents/nr-reporting-summary-flat.pdf

Life sciences study design

All studies must disclose on these points even when the disclosure is negative.

Sample size	The chosen sample size are based on the numbers used for previous publications (PMID: 30029001; PMID: 31447391), which is most optimal to generate statistically significant results. No statistical methods were used to predetermine sample sizes.
Data exclusions	No samples or animals were excluded from the analyses.
Replication	All replicates are biological replicates obtained from biologically independent experiments. All attempts at replication were successful. The experiments number has been clearly stated in the figure legends.
Randomization	The samples/cells were randomized to be examined. The mice were randomized to put into separate groups /cages for experiments.
Blinding	For all experiements, the investigators were divided into two groups. One group were blinded to allocation during experiments and outcome assessment.

Reporting for specific materials, systems and methods

We require information from authors about some types of materials, experimental systems and methods used in many studies. Here, indicate whether each material, system or method listed is relevant to your study. If you are not sure if a list item applies to your research, read the appropriate section before selecting a response.

Materials & experimental systems

n/a	Involved in the study
☐	☒ Antibodies
☐	☒ Eukaryotic cell lines
☒	☐ Palaeontology and archaeology
☐	☒ Animals and other organisms
☐	☒ Clinical data
☒	☐ Dual use research of concern
☒	☐ Plants

Methods

n/a	Involved in the study
☒	☐ ChIP-seq
☒	☐ Flow cytometry
☒	☐ MRI-based neuroimaging

Antibodies

Antibodies used	Rabbit monoclonal antibodies against HA (3724S), phospho-MEK1/2 (9154S), MEK1/2 (8727S), phospho-AKT (4056S), STAT3 (63585S), phospho-EGFR Y845 (6963S), phospho-EGFR Y1173 (4407S), EGFR (4267S) and rabbit polyclonal antibodies against phospho-EGFR Y992 (2235S), phospho-EGFR Y1045 (2237S), phospho-EGFR Y1086 (2220S), phospho-EGFR Y1148 (4404S), and phospho-EGFR Y1068 (2234S) were obtained from Cell Signaling Technology. Mouse monoclonal antibodies against GST (66001-2-Ig), His-tag (66005-1-Ig) and rabbit monoclonal antibody against Flag (20543-1-AP) were obtained from Proteintech Group. Mouse monoclonal antibody against phospho-ERK1/2 (sc-81492) and rabbit polyclonal antibody against ERK1/2 (sc-94) were purchased from Santa Cruz Biotechnology. Rabbit polyclonal antibodies against EGFR (A11351), Akt (A11016) and phospho-STAT3 (AP0070) were brought from Abclonal Technology. Mouse monoclonal antibody against Tubulin (T5201) was purchased from MilliporeSigma. The following secondary antibodies were used: goat-anti-mouse IgG second antibody (31160, Thermo); goat-anti-rabbit IgG second antibody (31210, Thermo). The primary antibodies were used at a 1:1000 dilution and the secondary antibodies were used at 1:3,000 dilution for immunoblotting.
Validation	All the antibodies were validated by manufacturers for indicated species and applications.

Eukaryotic cell lines

Policy information about cell lines and Sex and Gender in Research

Cell line source(s)	HEK293T (GNHu17), H1299 (TCHu160), U87 (TCHu138), and U251 (TCHu 58) were obtained from the cell library of the Chinese Academy of Science. Glioma stem cell line GSC387 was a gift from Prof. Huairui Yuan at CAS Center for Excellence in Molecular Cell Science.
Authentication	Cells were authenticated using the short tandem repeat (STR) method.
Mycoplasma contamination	All cell lines were routinely tested negative for mycoplasma contamination.
Commonly misidentified lines (See ICLAC register)	No commonly misidentified cell lines were used.

Animals and other research organisms

Policy information about studies involving animals; ARRIVE guidelines recommended for reporting animal research, and Sex and Gender in Research

Laboratory animals	BALB/c nude mice (female, 6 weeks) were purchased from SLAC Laboratory Animal Co., Ltd. (Shanghai, China).
Wild animals	The study did not involve wild animals.
Reporting on sex	All experiments were conducted with female mice to limit complications of male territorial behavior and fighting during long-term cancer experiments.
Field-collected samples	The study did not involve samples collected from the field.
Ethics oversight	All animal experiments were approved by the Institutional Animal Care and Use Committee (IACUC) of Shanghai Institute of Biochemistry and Cell Biology (approval number: SIBCB-S355-2312-40), Chinese Academy of Sciences and complied with all relevant ethical regulations.

Note that full information on the approval of the study protocol must also be provided in the manuscript.

Clinical data

Policy information about clinical studies

All manuscripts should comply with the ICMJE guidelines for publication of clinical research and a completed CONSORT checklist must be included with all submissions.

Clinical trial registration	Not applicable.

Study protocol	Not applicable.
Data collection	Not applicable.
Outcomes	Not applicable.

Plants

Seed stocks	*Report on the source of all seed stocks or other plant material used. If applicable, state the seed stock centre and catalogue number. If plant specimens were collected from the field, describe the collection location, date and sampling procedures.*
Novel plant genotypes	*Describe the methods by which all novel plant genotypes were produced. This includes those generated by transgenic approaches, gene editing, chemical/radiation-based mutagenesis and hybridization. For transgenic lines, describe the transformation method, the number of independent lines analyzed and the generation upon which experiments were performed. For gene-edited lines, describe the editor used, the endogenous sequence targeted for editing, the targeting guide RNA sequence (if applicable) and how the editor was applied.*
Authentication	*Describe any authentication procedures for each seed stock used or novel genotype generated. Describe any experiments used to assess the effect of a mutation and, where applicable, how potential secondary effects (e.g. second site T-DNA insertions, mosiacism, off-target gene editing) were examined.*
